# Narcissistic susceptibility to conspiracy beliefs exaggerated by education, reduced by cognitive reflection

**DOI:** 10.3389/fpsyg.2023.1164725

**Published:** 2023-07-06

**Authors:** Tylor J. Cosgrove, Christopher P. Murphy

**Affiliations:** Department of Psychology, Faculty of Society and Design, Bond University, Gold Coast, QLD, Australia

**Keywords:** narcissism, uniqueness, supremacy, collective narcissism, conspiracy theories, education, critical thinking

## Abstract

Conspiracy theories are alternate viewpoints of provided explanations; sensational stories revolving around small groups exerting control for nefarious reasons. Recent events and research have outlined myriad negative social and personal outcomes for those who endorse them. Prior research suggests several predictors of susceptibility to conspiracy theories, including narcissistic personality traits (grandiosity, need for uniqueness), cognitive processes (critical thinking, confirmation bias) and lack of education. The aim of the current paper was to explore how facets of narcissism predict susceptibility to conspiracy theories. It was expected that narcissism would be a positive predictor, but education and cognitive reflection would act as protective factors, reducing this effect. Study one utilized an international survey (*N* = 323) to investigate the role of education as a protective tool in the relationship between narcissistic traits and conspiratorial beliefs. Support was found for the hypotheses that individuals with higher levels of grandiosity, vulnerable narcissism, a strive for uniqueness, and a strive for supremacy predicted higher levels of conspiracy endorsement. Higher education and STEM education were associated with lower levels of conspiracy endorsement, however all significant moderations indicated that for narcissistic individuals, education increased their likelihood of adopting conspiracy beliefs, contrary to expectation. To investigate this further, study two analyzed a large-scale publicly available dataset (*N* = 51,404) to assess the relationship between narcissism, critical thinking skills (specifically cognitive reflection) and conspiracy beliefs pertaining to the COVID-19 pandemic. As expected, analysis found narcissism and poor cognitive reflection (intuitive thinking) as predictors of conspiracy beliefs. Higher levels of cognitive reflection were found to be protective, moderating and reducing the impact of narcissism on endorsement of conspiracy theories. The findings suggest that cognitive reflection, but not education protect against narcissistic conspiracy belief. Moreover, that cognitive reflection may have a lessened effect against conspiracy theories adopted for social or ideological reasons. These findings improve understanding of both the role and limitations of education/critical thinking skills as protective factors against conspiracy theory endorsement.

## Introduction

‘Conspiracy theories’ are viewpoints that are negatively toned, revolving around malfeasance within a variety of higher power constructs (Napolitano and Reuter, 2021). Recently, there has been a notable surge in interest and research dedicated to the study of conspiracy theories and individuals who hold such beliefs, revealing a range of adverse personal and societal consequences associated with their adoption (Gerts et al., 2021; Wang and Kim, 2021). Consequently, inroads have been made into identifying the psychological processes and predictors of conspiracy theory belief, as well as factors that may mitigate these mechanisms.

Personality traits have been suggested to influence an individual’s willingness to endorse conspiracy theories, including anxiety, trust and dark triad traits. Narcissism has been found to be a particularly robust predictor, due to its ability to explain considerable variance in conspiracy theory endorsement (Cichocka et al., 2016; Hughes and Machan, 2021; Sternisko et al., 2021; Stasielowicz, 2022). Research shows that narcissistic individuals often fall prey to dichotomous thinking; they prefer to deal in absolutes, viewing issues as ‘black or white’ (Oshio, 2012; Jonason et al., 2018). Conspiracy theories are often described to provide simple explanations for complex issues, and often find broader appeal in times of uncertainty, such as times of war, widespread disease, natural disasters and terrorism (van Prooijen and Douglas, 2017; Douglas et al., 2019). This reduction in uncertainty has been referred to as an ‘existential’ motivation for conspiracy theory adoption (Douglas et al., 2017). As such, conceptual sense can be made of why narcissistic individuals find appeal in the reductive explanations offered by many conspiracy theories.

Narcissism has also been linked to low levels of ‘intellectual humility’ (Krumrei-Mancuso et al., 2019; Bak and Kutnik, 2021). This indicates that narcissistic individuals are more likely to overestimate the accuracy of their own beliefs, and resist adjusting them in the face of conflicting information. Low intellectual humility has recently been identified as a predictor of susceptibility to fake news, pseudoscientific beliefs and conspiratorial ideation (Koetke et al., 2021; Bowes and Tasimi, 2022). This strong resistance to information that challenges existing beliefs and accepting conspiratorial information that supports current belief is referred to as the ‘epistemic’ motivation for conspiracy theory adoption (Douglas et al., 2017). A positive relationship between narcissism and ‘counterfactual’ thinking, and a negative relationship between narcissism and critical thinking skills like cognitive reflection have also been established (Littrell et al., 2020; Grundmann et al., 2022). In this study we explore specific factors that may mitigate these mechanisms, including education and critical thinking.

### Education and critical thinking skills

In the literature of reducing conspiracy theory adoption, key factors identified are greater education and critical thinking skills (van Prooijen, 2017; De Coninck et al., 2021; Lantian et al., 2021). Several studies have established connections between higher levels of education and intelligence, cognitive ability and critical thinking skills (Brinch and Galloway, 2012; Dunne, 2015; Ritchie and Tucker-Drob, 2018). Moreover, a link between lower levels of education and conspiracy theory endorsement is also well established (Douglas et al., 2016; van Prooijen, 2017; Georgiou et al., 2020; Knobel et al., 2022). However, the role of education and critical thinking skills such as cognitive reflection remain unexplored as protective factors in the personality, conspiracy belief relationship.

In investigating the link between education and conspiracy belief, van Prooijen (2017) tested mediation models to see which factors might explain the relationship. They found a dual mediation model to be significant in which analytic thinking and decreased belief in simple solutions mediated the relationship between education and conspiracy belief. Although historically the causality of education on cognitive ability has been challenged, recent research has rebutted the notion and found strong support for a causal link (Brinch and Galloway, 2012; Ritchie and Tucker-Drob, 2018). A relationship between STEM (Science, Technology, Engineering, and Mathematics) education and decreased conspiracy belief has also been established, thought to be explained at least partially explained by pro-science outlook and critical thinking ability (Savran Gencer and Doğan, 2020; Pavic and Suljok, 2022). These findings strongly suggest that education may protect against the link between the narcissistic desire for simplicity and conspiracy theory endorsement. As such, the current paper will focus on whether education and critical thinking skills mitigate the link between narcissism and conspiracy belief, and whether each facet of narcissism show differences in efficacy.

### Grandiose/vulnerable narcissism

In the literature, grandiosity has historically been the hallmark of narcissism. It refers to a heightened self-image, a sense of entitlement, as well as manipulative and aggressive tendencies (Miller et al., 2011). Studies consistently show a link between grandiose narcissism and both generic conspiracy theory endorsement and COVID-19 specific conspiracy theory endorsement (Kay, 2021; Ük and Bahcekapili, 2022). Vulnerable narcissism shares core characteristics with grandiose narcissism of antagonism and entitlement, however is differentiated by emotional dysregulation, insecurity, low self-esteem, and heightened self-consciousness (Miller et al., 2010; Kay, 2021). There has been limited research into the relationship between vulnerable narcissism and conspiracy theory endorsement, however current perspective is that their antagonistic, untrusting, and paranoid nature contributes to a predisposition to conspiracy theory endorsement (Kay, 2021; Cichocka et al., 2022).

### Narcissistic need for uniqueness/superiority

A more recent division of narcissism that has been discussed in the context of conspiracy theory adoption is a need for both uniqueness and superiority among others. These two needs align with the ‘social motivation’ of Douglas et al. (2017). Conspiracy theories allow the adopter to feel privy to information that others are not and that they are morally righteous relative to the shadowy out-group supported by most conspiracy narratives. The rhetoric around conspiracy theories also often includes the notion of adopters being ‘awake’ or ‘enlightened.’ As such, narcissistic individuals may adopt conspiracy theories as a means of ‘standing out from the crowd’ and be perceived as unique, superior, important, and intelligent (Cichocka et al., 2016; Imhoff and Lamberty, 2017).

### Collective narcissism

Beyond simplifying complex issues and providing feelings of superiority, conspiracy theories have also been proposed to function as a means of supporting identity and ideology. These are thought to include signaling group membership, morally condemning out-groups, and satisfying needs for belonging (Douglas et al., 2017; Robertson et al., 2022). These aspects of conspiracy theory belief have been attributed to facilitate increases in polarization, conflict, and intergroup prejudice (Enders and Smallpage, 2018; Jolley et al., 2020). This socially driven motivation may help to explain findings that narcissism at the group level (collective narcissism) has also been found to be robust predictor of conspiracy belief (Golec de Zavala et al., 2022). Collective narcissism refers to the phenomenon of a group of individuals feeling as though their group of membership is superior to others and that they deserve recognition (de Zavala et al., 2009). It is unclear whether education and cognitive abilities protect against conspiracy theory adoption as means of signaling ideology or identity, however they will be assessed in the current study.

### Aims, rationale and hypotheses

Extensive literature has established the link between narcissism and conspiracy beliefs and as such we expect that all facets of narcissism measured will positively predict conspiracy belief. To our knowledge, the role of education and associated cognitive abilities has not been assessed in this context. As such, the research aims to investigate the moderating ability of level and type of education in the relationship between different facets of narcissism and conspiracy theory endorsement. Considering substantial exposure during education to critical thinking, evidence appraisal and the scientific method, we expect higher levels of education, and specifically STEM-related education to protect against the dichotomous worldview associated with narcissism. Specifically, we expect these factors to moderate the relationships, reducing or reversing the effect. Furthermore, we consider that education and associated positive outcomes may work to satisfy needs for feelings of uniqueness and superiority, thus reducing the likelihood of conspiracy theory adoption. To further investigate the link between narcissism and conspiracy theory adoption, in study two we will analyze a publicly available dataset (Azevedo et al., 2022) to assess the impact of critical thinking skills on these relationships. Aligned with expectations of education, we expect increased critical thinking skills to reduce the impact narcissism has on likelihood of conspiracy theory adoption.

## Study one

### Method

#### Participants

Participants were recruited via Prolific.co. Funding was provided by Bond University. Demographic information was collected as a requirement of the survey. This included the participants age, gender, ethnicity, highest education achieved, area of education (Table 1).

**Table 1:** Breakdown of participants by demographics.

Category	Levels	*N*	%
Age	18–25	55	17.1
	25–35	110	34.1
	35–45	73	22.7
	45+	43	13.4
	55+	41	12.7
Gender	Female	156	48.6
	Male	163	50.8
	Non-Binary	3	0.9
Education	High School	78	24.1
	Certificate/Diploma	69	21.4
	Bachelor’s Degree	114	35.3
	Post-Graduate/Honors	19	5.9
	Master’s Degree	34	10.5
	Doctorate/Post-Doctorate	9	2.8
Country of residence	Australia	97	30
	United Kingdom	196	59.5
	United States	34	10.5
Household income	Less than $10,000	21	6.5
	$10,000 – $19,999	24	7.4
	$20,000 – $29,999	38	11.8
	$30,000 – $39,999	30	9.3
	$40,000 – $49,999	50	15.5
	$50,000 – $59,999	21	6.5
	$60,000 – $69,999	18	5.6
	$70,000 – $79,999	28	8.7
	$80,000 – $89,999	14	4.3
	$90,000 – $99,999	19	5.
	$100,000 – $149,999	29	90
	$150,000 – $199,999	19	5.9
	$200,000 – $299,999	8	2.5
	$300,000+	4	1.2

*N* = 323.

#### Materials

Scales used to measure personality traits and beliefs are displayed in Table 2. The table includes number of items, scale type, means, standard deviations and Cronbach’s alphas. Datasets including all items used across both studies and R code used for analyses are available at https://osf.io/tydur/.

**Table 2:** Psychometric tools and descriptives.

Tool	*M*	*SD*	Cronbach’s α	Items/Scale
Generic Conspiracy Belief Scale (GCBS) (Brotherton et al., 2013)	2.57	0.91	0.95	15 items 5-point Likert Scale
Narcissistic Admiration/Rivalry Questionnaire (NARQ) (Back et al., 2013)	2.45	0.76	0.80	18 Items 6-point Likert Scale
NARQ – Grandiosity	2.58	1.04	0.67	3 Item subscale 6-point Likert Scale
NARQ – Need for Uniqueness	2.90	1.10	0.75	3 Item subscale 6-point Likert Scale
NARQ – Need for Supremacy	2.22	1.17	0.85	3 Item subscale 6-point Likert Scale
Hypersensitive Narcissism Scale (HSNS) (Hendin and Cheek, 1997)	2.96	0.60	0.72	10 Items 5-point Likert Scale
Collective Narcissism Scale (CNS) (de Zavala et al., 2009)	2.51	1.04	0.84	5 items 6-point Likert Scale

### Procedure

Ethics approval was obtained from the Bond University Human Research Ethics Committee. The bulk of participants were recruited from Prolific.co (Australia, UK and the US) and a portion via the networks of the several investigators. Participants were able to complete the study from any location and only required an electronic device and internet access to complete the study. Attention checks were used in the form of a Likert style question prompting the participant to select a particular response. Participants who declined consent, failed attention checks or completed the survey in a time deemed unlikely to be genuine responses (<10 min) were not included in data analysis.

### Results

**Figure 1:**
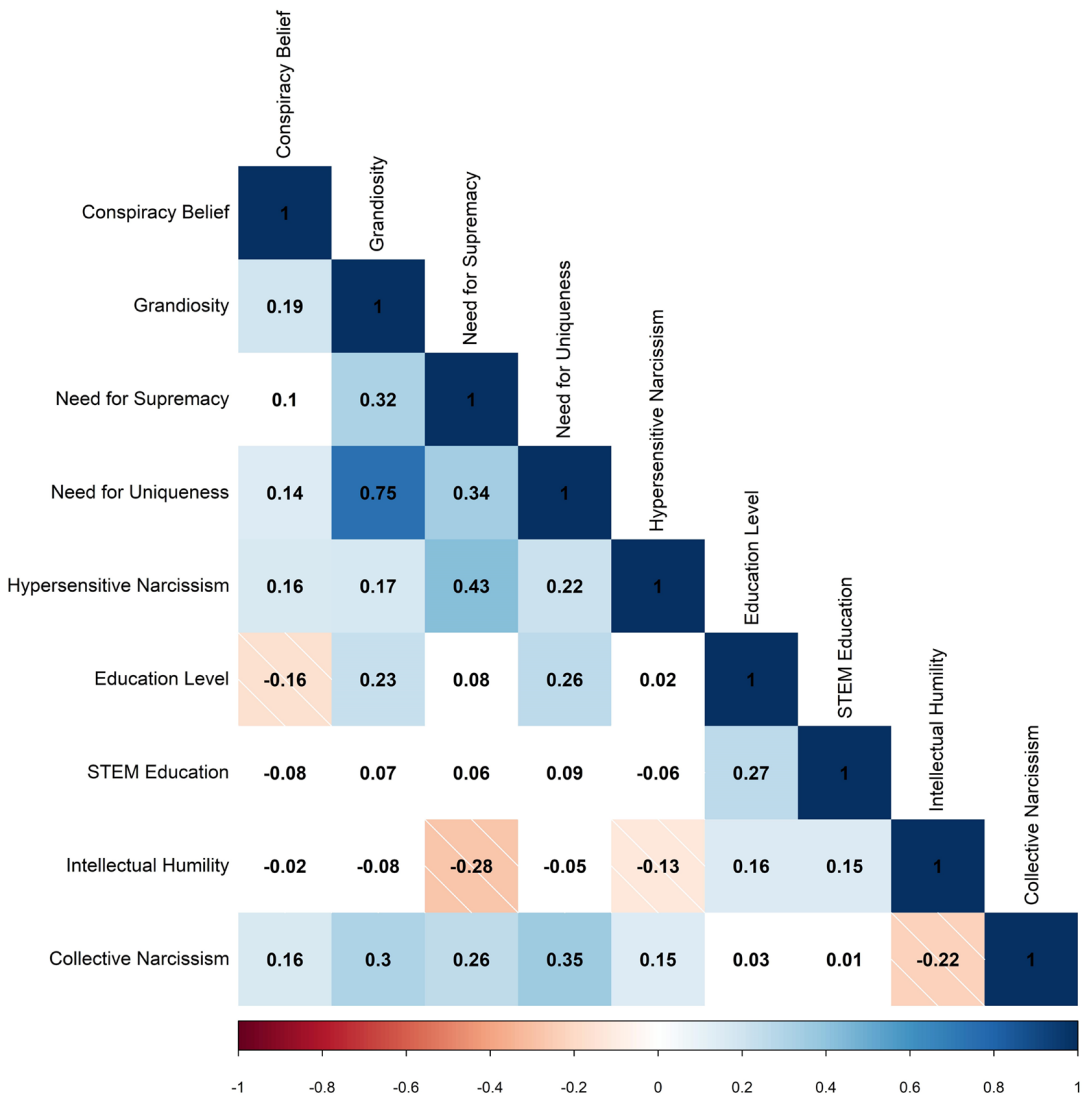
Pearson’s correlations for study one factors. *N* = 254, unshaded boxes indicate non-significant correlation.

Prior to analysis, data was cleaned, and area of education was collaboratively filtered by the authors. Examples of STEM areas of education were “physics,” “medicine,” “information technology,” “computer science,” “engineering,” “mathematics” and “statistics.” Due to the skewed nature of data regarding narcissism in the general population, outliers were Winsorized to minimize undue influence on results while minimizing loss of information. Below are factor correlations and moderated regression plots. The data was analyzed in R version 4.2.2 and plots were produced with the ‘corrplot’ and ‘plotmod’ packages. After removing cases with missing values, study one contained 254 participants and study two contained 45,210 for analysis. See Figure 1 for study one variable correlations. Regression analyses were performed both with and without controlling for political ideology. In study one, there was no significant relationship between political orientation and conspiracy belief. In study two, self-reported conservatism was positively correlated with higher belief in COVID-related conspiracy, however after controlling for self-reported political orientation the interaction term showed a negligible change (standardized coefficients showed a difference of 0.004 and political orientation was subsequently left out of analyses).

### Regression analyses

**Figure 2:**
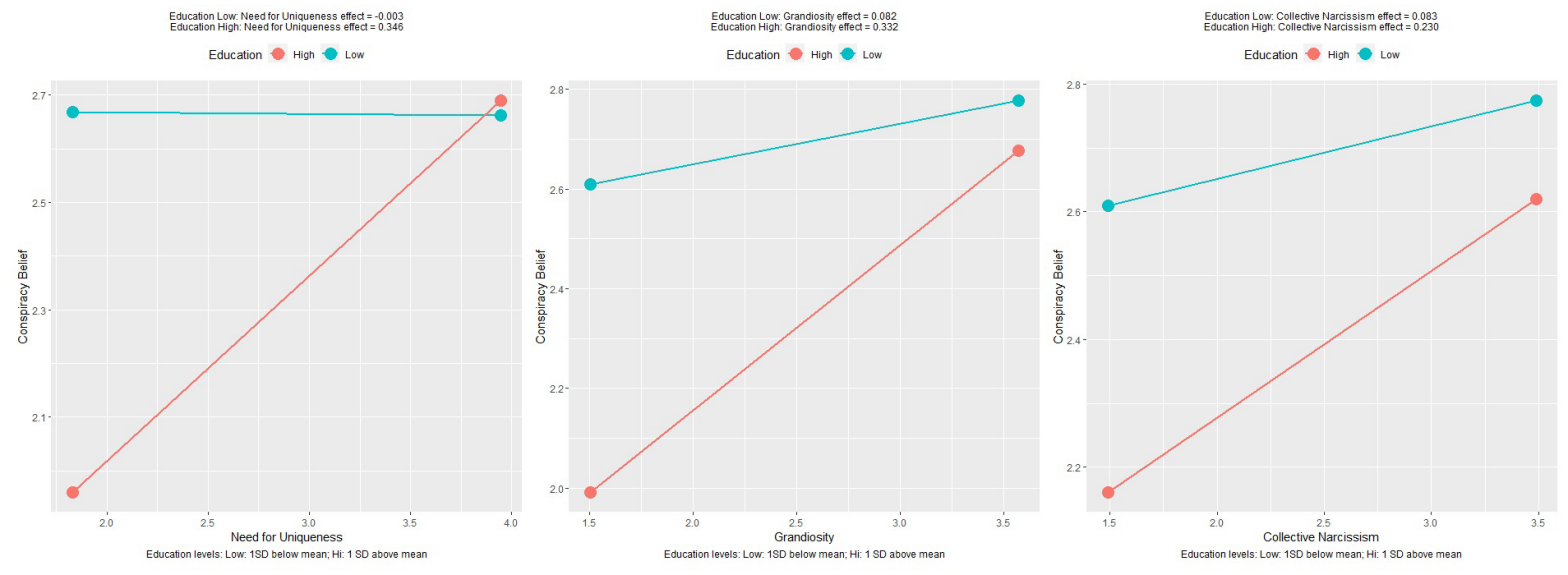
Plots of relationships between facets of narcissism and conspiracy belief, moderated by education.

A series of regressions were performed to assess how well narcissistic facets, hypersensitive narcissism and collective narcissism predicted endorsement of generic conspiracy beliefs, as measured by the GCBS. Moderated regressions were performed to assess whether education would interact to exacerbate or reduce these effects. Need for uniqueness was a significant positive predictor of conspiracy belief, *F*(1,250) = 5.25, *p* = 0.023 and accounted for 2.2% of variance. The education interaction term also contributed significantly to the model, *F*(3,250) = 8.31, *p* < 0.001 with the model accounting for 9.1% of variance in conspiracy belief. The positive interaction coefficient indicates education strengthened the relationship between need for uniqueness and conspiracy belief, as shown in Figure 2. The STEM interaction term did not significantly contribute to the model, indicating STEM education did not change the relationship between need for uniqueness and conspiracy belief.

**Figure 3:**
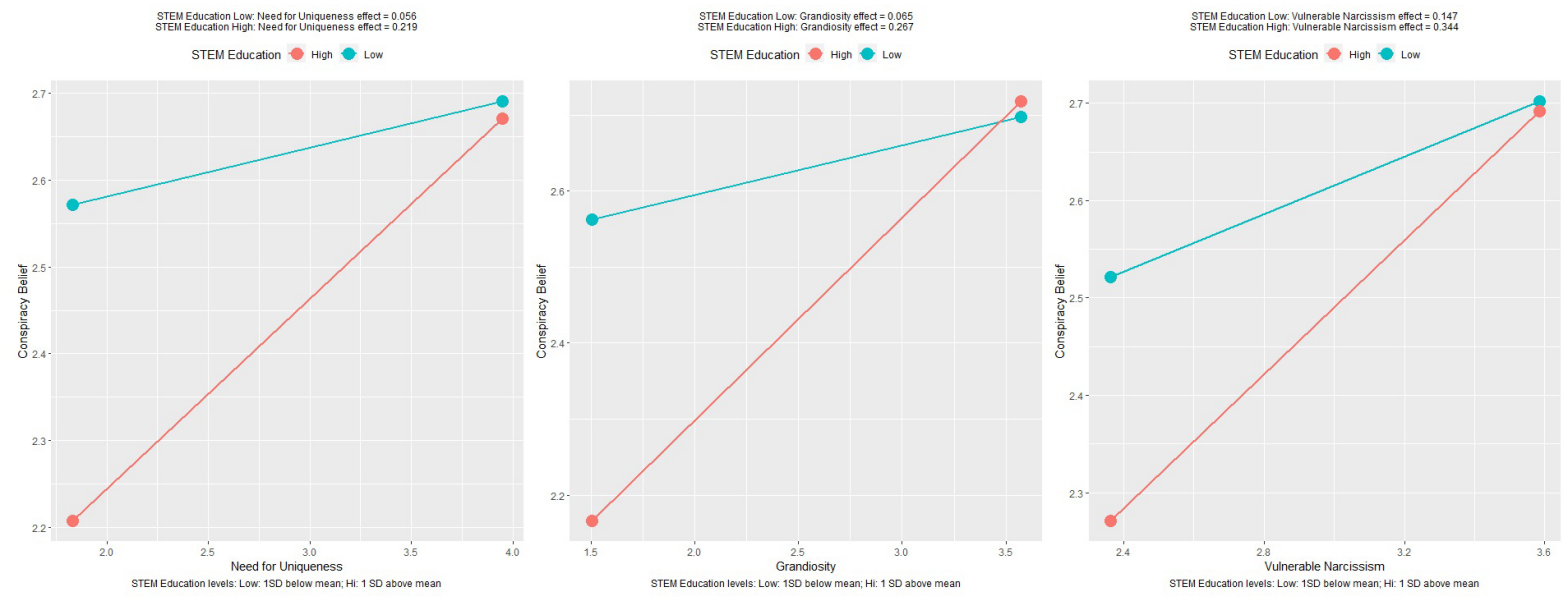
Plots of relationships between facets of narcissism and conspiracy belief, moderated by STEM education. Please note these interactions are not significant, plots are for observing trends only.

Grandiosity was a significant positive predictor of conspiracy belief, *F*(1,252) = 9.93, *p* = 0.002 and accounted for 3.7% of variance. The education interaction term also contributed significantly to the model, *F*(3,250) = 8.86, *p* < 0.001 with the model accounting for 9.6% of variance in conspiracy belief. The positive interaction coefficient indicates education strengthened the relationship between grandiosity and conspiracy belief, as shown in Figure 2. The STEM interaction term did not significantly contribute to the model, however non significant interactions are shown in Figure 3.

Need for supremacy was a significant positive predictor of conspiracy belief, *F*(1,252) = 9.93, *p* = 0.002 and accounted for 3.7% of variance. The education interaction term also contributed significantly to the model, *F*(3,250) = 8.86, *p* < 0.001 with the model accounting for 9.6% of variance in conspiracy belief. The positive interaction coefficient indicates education strengthened the relationship between a need for supremacy and conspiracy belief, as shown in Figure 2. The STEM interaction term did not significantly contribute to the model.

Need for supremacy was not a significant predictor of conspiracy belief. The addition of both the education and STEM interaction terms did not contribute significantly to the model. Vulnerable narcissism was found to be a significant positive predictor of conspiracy belief, *F*(1,252) = 6.63, *p* = 0.011, accounting for 2.6% of variance. The addition of the education and STEM interaction terms did not explain a significant amount of additional variance. This indicates higher education, nor STEM education impacted the relationship between vulnerable narcissism and conspiracy belief. Lastly, collective narcissism was also found to be a significant predictor of conspiracy belief, *F*(1,252) = 6.96, *p* = 0.008, accounting for 2.6% of variance. Again, the addition of neither of the interaction terms accounted for a significant amount of additional variance, indicating the relationship was stable regardless of education. See Table 3 for coefficients with significance and Figure 2 for significant plots.

**Table 3:** Coefficients and significance of regressions for conspiracy belief (study one).

Predictor	*β*	Education interaction (*β*)	STEM interaction (*β*)
Education	−0.22***		
STEM-Education	−0.10		
Uniqueness	0.14*	0.18**	0.20
Grandiosity	0.18**	0.13*	0.24
Supremacy	0.08	0.00	−0.01
Vulnerable narcissism	0.16*	0.02	0.14
Collective narcissism	0.15**	0.07	0.02

***p < 0.001, **p < 0.01, *p < 0.05, Dependent variable: total score of generic conspiracy belief scale.

## Study two

A publicly available dataset that was collected by a team of ~200 researchers during the COVID-19 pandemic was used to further investigate hypotheses (Azevedo et al., 2022). The data measures attitudes and behaviors regarding the pandemic and response as well as other psychosocial factors. As part of this survey, measures of narcissism and collective narcissism, a critical thinking task, and several common conspiracy theories regarding COVID-19 were included. Education level or field was not recorded as part of the study.

### Results

**Figure 4:**
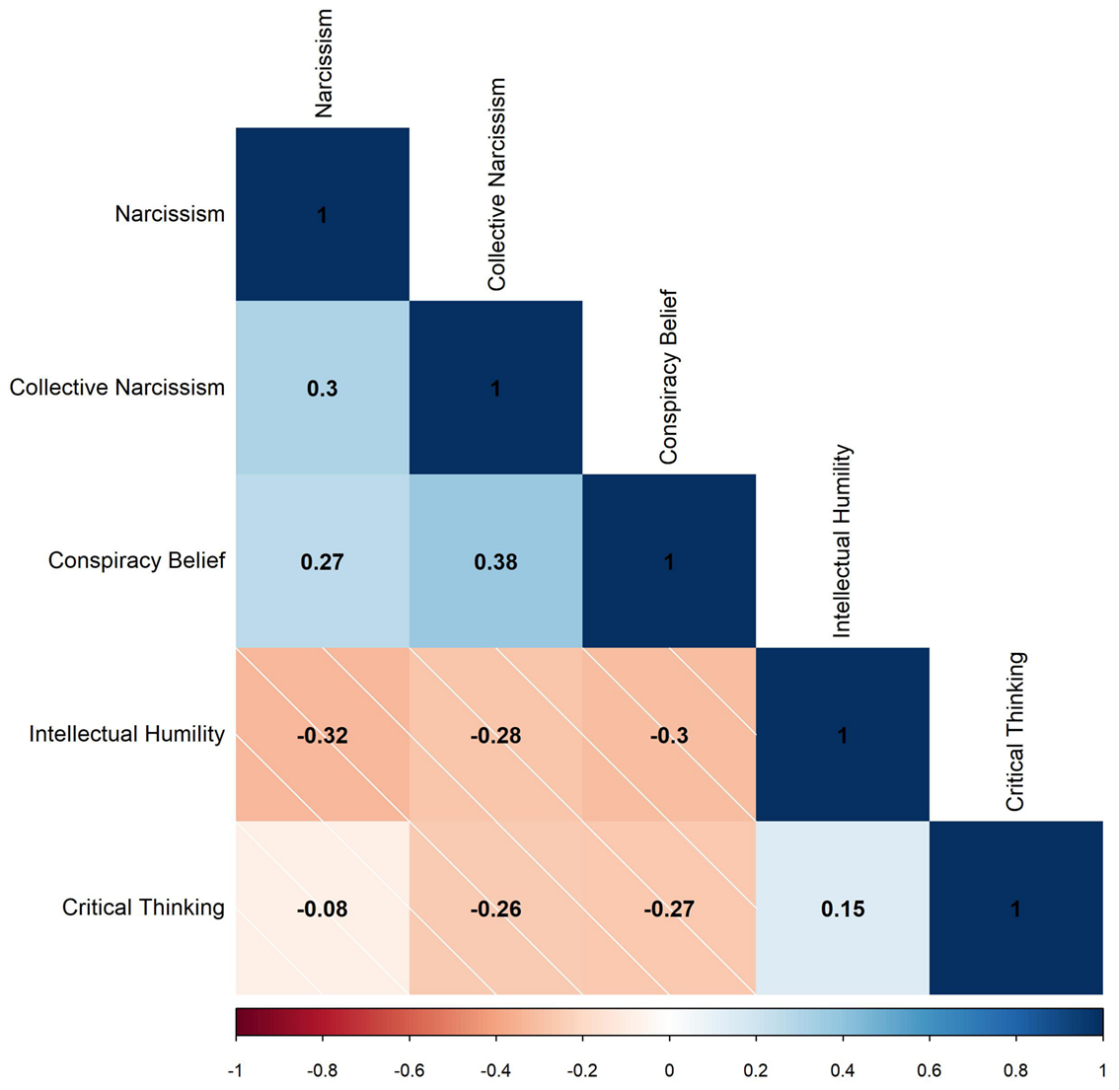
Pearson’s correlations for study two factors. *N* = 45,210, blank cells indicate no significant correlation.

**Figure 5:**
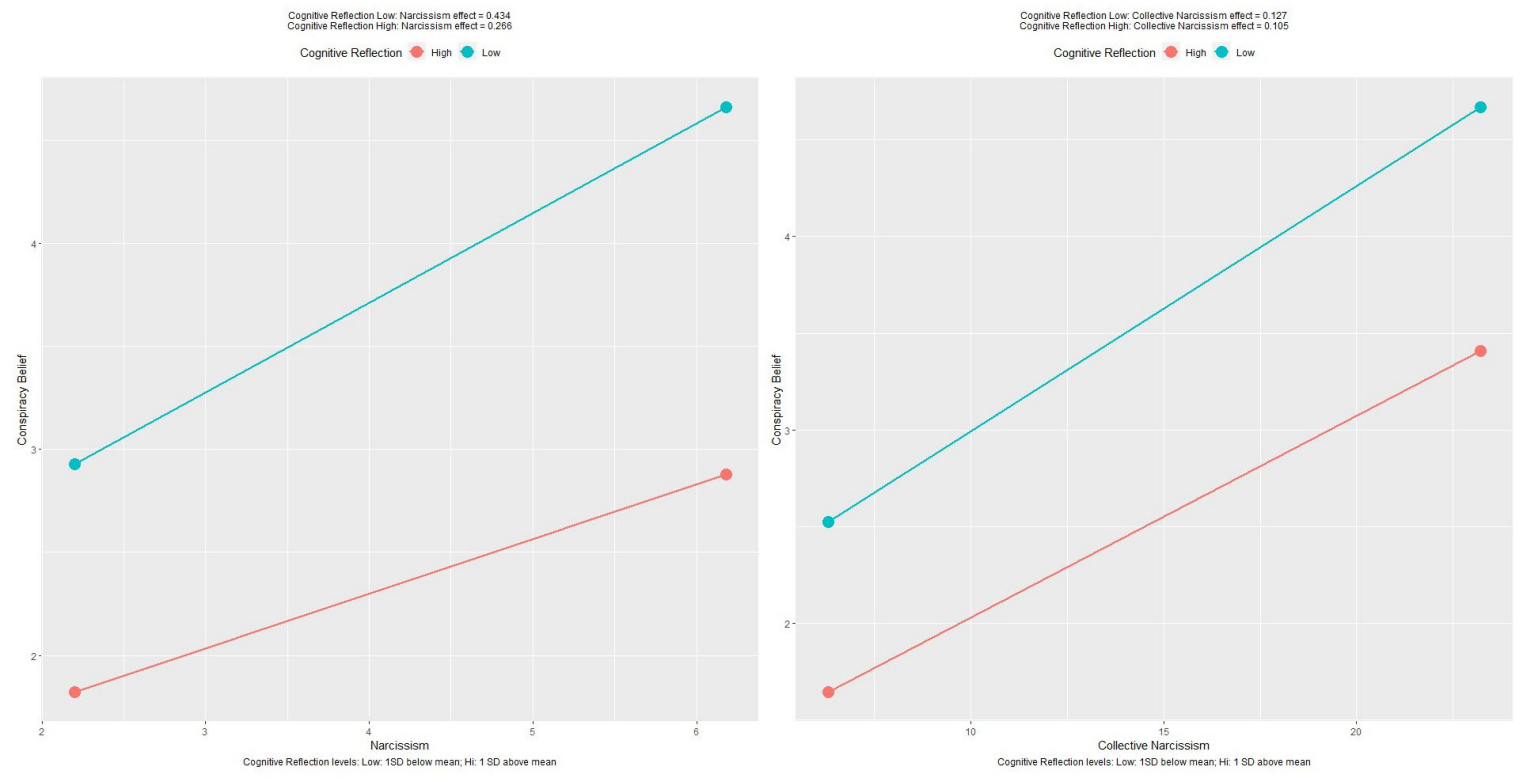
Plots of relationships between individual and collective narcissism, moderated by cognitive reflection.

The publicly available dataset was used to further explore the relationships between narcissism and conspiracy belief, with regard to critical thinking as a moderator. Correlations are shown below in Figure 4, regression coefficients with significance are shown in Table 4 and moderated regression plots are shown in Figure 5.

**Table 4:** Coefficients and significance of regressions on conspiracy belief (study two).

Predictor	*β*	Cognitive reflection interaction (β)
Narcissism	0.27***	−0.11***
Collective narcissism	0.36***	−0.06***

***p < 0.001. Dependent variable: total score on conspiracy theories related to COVID-19.

Regression analyses were performed to assess the predictive ability of narcissism and collective narcissism on endorsement of conspiracy theories regarding COVID-19. Moderated regressions aimed to assess whether the critical thinking skill of cognitive reflection would interact and reduce the relationship. Narcissism was a significant positive predictor of COVID-related conspiracy belief, *F*(1,48470) = 3738, *p* < 0.001 and accounted for 7.1% of variance. The cognitive reflection interaction term also contributed significantly to the model, *F*(3,48468) = 2494, *p* < 0.001 with the model accounting for 13.4% of variance in conspiracy belief. The negative interaction coefficient indicates cognitive reflection was protective, weakening the relationship between narcissism and COVID conspiracy beliefs. Collective narcissism was also a significant positive predictor of COVID-related conspiracy belief, *F*(3,48470) = 8000, *p* < 0.001 and accounted for 14.3% of variance. The cognitive reflection interaction term also contributed significantly to the model, *F*(3,48468) = 3378, *p* < 0.001 with the model accounting for 17.5% of variance in conspiracy belief. The negative interaction coefficient indicates cognitive reflection was again protective, weakening the relationship between collective narcissism and COVID-related conspiracy beliefs. Interactions can be seen below in Figure 5.

## Discussion

This study aimed to expand on the growing body of research exploring the relationship between different narcissistic traits and adoption of conspiracy beliefs, with specific regard to education and cognitive reflection as protective factors. As expected, and mostly in accordance with previous literature, all facets of narcissism at the individual and group level predict increased endorsement of generic and COVID-19 specific conspiracy theories across two studies, with the exception of ‘need for supremacy.’

As expected, higher education, STEM education and cognitive reflection skills were all associated with lower endorsement of conspiracy theories across the two studies. The moderated regressions performed revealed unexpected and interesting results. As shown in figures two, three & five, the relationships between narcissistic facets and conspiracy theories were differentially impacted by education level/field and cognitive reflection skills. Most moderations were not significant, and those that show significance are in contrast with the expected findings. The relationships that were moderated by level of education or STEM education in study one showed an increase in the effect of narcissism on conspiracy theory belief. These interactions suggest that the protective effect of education is only true in the absence of higher levels of narcissistic traits.

The findings of study one suggest that when narcissistic traits are high, increased education either exacerbates the effect, or has no effect at all, depending on the facet of narcissism. Analysis suggested that those who have high levels of overall narcissism, grandiosity and need for uniqueness were more likely to endorse conspiracy theories, and this effect was stronger when these individuals had obtained higher levels of education, a finding that is in stark contrast to the hypothesized impact of education on this relationship. The other main finding from analyses is the significant moderation of cognitive reflection on the relationship between narcissism and COVID-19 conspiracy theories in study two. These findings were in line with the hypothesized effect, that cognitive reflection skills interact with narcissism to reduce the effect on conspiracy theory endorsement. The interaction was also significant for collective narcissism, likely due to large sample size, however the effect was modest in comparison.

These findings may be explained by several processes. Firstly, it may be that narcissistic individuals may experience increased confidence in their own opinions and beliefs when they are also highly educated. Higher levels of education may increase perceived expertise, exponentially decreasing the already low levels of intellectual humility seen in narcissistic individuals (Bak and Kutnik, 2021). Intellectual arrogance is the antithesis of cognitive reflection, and as such might partially explain adoption of pseudoscientific, unfounded, and conspiratorial beliefs. Although many conspiracy theorists claim to be ‘sceptics’, they often do not appear to apply the same skepticism to their own beliefs. Indeed, a key part of holding rational beliefs is being willing to both challenge and reassess them, which may be inconducive to the narcissistic worldview. Considering this is a desired outcome of higher education, it appears that those high in narcissism may be resistant to adopting such cognitive features and thus their absence plays a role in forming alternative beliefs.

Another possible explanation is that of the counterintuitive effect of education and science literacy on belief. A seminal study by Kahan et al. (2012) suggested that those with the highest level of scientific literacy, were most polarized in their opinions regarding contentious topics such as evolution and climate change. They posit that when motivated by ideology or identity, those with scientific training may employ their reasoning skills to protect and support existing beliefs, rather than engage in Bayesian belief updating. This notion may explain the unexpected interaction effects in the current study. Narcissistic individuals are drawn to conspiracy theories for several reasons, including their tendency to prefer simple explanations and wishing to be seen as unique and better than others (Cichocka et al., 2022). These may be key parts of their identity, of which narcissists may be defensive of, as is part of their self-preserving nature. As such, when narcissistic individuals also attain higher education, they may recruit these reasoning abilities to bolster their pre-existing tendency to conspiratorial explanations. This is also evident in the pseudo-scientific approach often seen in attempts to support conspiracy theories, e.g., sightings of tall buildings in distant cities, or flight paths deviating from a straight line as evidence that the earth is flat.

Although findings from study two seem contradictory, given the causal link well established between education and critical thinking, these findings may also be explained by established relationships between narcissism and cognition. Littrell et al. (2020) find that narcissistic individuals tend to perform poorly in cognitive reflection tasks, moreover, Christopher et al. (2021) found that those that narcissistic individuals were significantly more likely to show signs of the Dunning-Kruger effect in the cognitive reflection task. Taken together with the current findings, narcissistic individuals are much more likely to favor intuitive thinking, and to be overconfident in their reasoning abilities. This mechanism may explain the differences in impact that education and cognitive reflection have on the narcissism, conspiracy belief relationship.

From the findings, it appears that some differences may also differ for the various facets of narcissism. In study one, the significant interactions are mostly present for facets of narcissism associated with the grandiose style, whereas the adversarial need for supremacy/collective narcissism did not see a significant interaction, nor did vulnerable narcissism. This further suggests that the interaction of education to exacerbate conspiracy beliefs likely revolves around an overconfidence in assessing information and inflated value of one’s own beliefs and opinions, as are characteristics of the grandiose style. This notion is supported by previous findings that those high in grandiose narcissism may be prone to gullibility (Hart et al., 2021).

This notion finds similar support in the moderate impact of cognitive reflection skills on the link between collective narcissism and conspiracy beliefs, when compared to individual narcissism. This suggests a differential impact of education and cognitive reflection skills depending on how individuals have arrived at conspiratorial beliefs. In line with the social motivation proposed by Douglas et al. (2017), individuals often adopt conspiracy theories to bolster or signal their group memberships and associated ideologies. As such, the findings suggest that conspiratorial beliefs arrived at through social or ideological motivations, rather than cognitive biases or logical fallacies may be more impervious to the positive effects of cognitive reflection.

### Limitations

The current study has certain limitations. Measurement of traits that are not normally distributed in the general population create some difficulties in collecting and analyzing data. Dark triad traits such as narcissism often effect skewed data due to the limited number of individuals that score highly and the majority who generally have low levels and to remove all outliers is to remove all individuals of interest. In the current study, Windsorizing of data reduced skewness to an acceptable level while including the individuals of interest. The use of subscales to measure narcissistic traits, such as the need for uniqueness and grandiosity, may impact the validity of the findings. These subscales, although previously validated and demonstrating acceptable internal consistency in the current study, have a small number of items. However, previous studies have identified them as central components of narcissism, showing good convergent validity (Cichocka et al., 2022; Jordan et al., 2022). Future studies may wish to confirm the current findings using a dedicated measure of grandiosity. Using data from different datasets allowed for a more robust assessment, however may pose difficulties as the same measures were not used across both studies, and the outcome variables were related to generic conspiracy theories and COVID-19 specific conspiracy theories, respectively. It is recommended that this be explored in a more controlled manner in future studies.

## Conclusion

The findings discussed in the current study add to the growing understanding of the link between conspiracy theories and one of the most robust predictors of their adoption, narcissism. It appears from the findings across the two studies that although education plays an important protective role against conspiracy theory adoption for the general population, this effect does not appear to be evident for narcissistic individuals.

Based on the current findings, it is indicated that individuals with narcissistic tendencies may exhibit limited involvement in Bayesian reasoning, which involves the critical evaluation of information and the adjustment of beliefs based on new evidence. The results suggest that educational attainment and scientific literacy might not exert the intended influence on such individuals. Instead of engaging in neutral evaluation of evidence, narcissistic individuals are inclined to reinforce their own opinions, a tendency that aligns with their overconfident and defensive disposition.

To conclude, the findings from the current study help to explain the prominent connection between narcissism and conspiracy beliefs. They suggest that narcissistic individuals may be reluctant to engage in the cognitive processes that prevent the majority of individuals from adopting such beliefs. From the current analyses and previous literature, this appears likely to be explained by several factors, including a reluctance to consider information that does not align with existing opinions. Overconfidence is a hallmark of narcissists and conspiracy theorists alike (Light et al., 2022; Vranic et al., 2022). Both populations have a strong need to maintain their beliefs and may actively avoid or discount evidence that challenges their worldview, particularly when well educated. As such, strategies focused solely on education or providing accurate information (“debunking”) may not be sufficient. The current study’s findings suggest that interventions that target increasing cognitive reflection and intellectual humility may be more effective in reducing the relationship between narcissism and conspiracy beliefs.

Lastly, the narcissistic need for feelings of uniqueness and superiority also appears to partly explain the relationship with conspiracy beliefs. Narcissistic individuals may use conspiracy beliefs to boost their image of self and in-group. Embracing conspiracy beliefs can provide them with a sense of being part of an exclusive group that possesses special knowledge or insights that others are not privy to. Conspiracy theories are thought to also appeal to narcissistic individuals as a means of maintaining perceived victimhood of in-group and to justify moral condemnation of out-groups (Biddlestone et al., 2021). Therefore, efforts aimed at reducing conspiracy beliefs should address the underlying psychological factors that contribute to a victimized mentality, such as promoting empathy, compassion, and a sense of connectedness.

## Data availability statement

The datasets presented in this study can be found in online repositories. The names of the repository/repositories and accession number(s) can be found below: https://osf.io/tydur/.

## Ethics statement

The studies involving human participants were reviewed and approved by the Bond University Human Research Ethics Committee. The patients/participants provided their written informed consent to participate in this study.

## Author contributions

All authors were equally involved in the design of the study, collection of data, and writing of the manuscript.

## Funding

This research was funded by Bond University.

## Conflict of interest

The authors declare that the research was conducted in the absence of any commercial or financial relationships that could be construed as a potential conflict of interest.

## Publisher’s note

All claims expressed in this article are solely those of the authors and do not necessarily represent those of their affiliated organizations, or those of the publisher, the editors and the reviewers. Any product that may be evaluated in this article, or claim that may be made by its manufacturer, is not guaranteed or endorsed by the publisher.
